# Comparison of short-term peripheral nerve stimulation, single-target supraorbital nerve pulsed radiofrequency, and dual-target supraorbital nerve–stellate ganglion pulsed radiofrequency for herpes zoster ophthalmicus–associated pain: a retrospective cohort study

**DOI:** 10.3389/fnins.2026.1976324

**Published:** 2026-10-23

**Authors:** Chen Liang, Anxiang Liu, Wei Wang

**Affiliations:** 1 Department of Spine and Spinal Cord Surgery, Xuchang Central Hospital, Xuchang, China; 2 Graduate School, Beijing University of Chinese Medicine, Beijing, China; 3 Department of Pain Medicine, China–Japan Friendship Hospital, Beijing, China

**Keywords:** herpes zoster ophthalmicus, peripheral nerve stimulation, pulsed radiofrequency, stellate ganglion, zoster-associated pain

## Abstract

**Objective:**

To compare the clinical outcomes of short-term peripheral nerve stimulation (st-PNS), supraorbital nerve pulsed radiofrequency (SON-PRF), and combined supraorbital nerve and stellate ganglion pulsed radiofrequency (SON + SG-PRF) for herpes zoster ophthalmicus (HZO)-associated pain.

**Methods:**

This retrospective study included patients with HZO-associated pain who underwent st-PNS, SON-PRF, or SON + SG-PRF between January 2023 and January 2026. The primary outcome was the longitudinal change in Numerical Rating Scale (NRS) scores over 24 weeks. Secondary outcomes included the clinical response rate, anxiety (GAD-7), depression (PHQ-9), sleep quality (PSQI), and treatment-related adverse events. The incidence of postherpetic neuralgia (PHN) was assessed as an exploratory outcome only in patients with a disease duration of ≤90 days at treatment. Longitudinal outcomes were analyzed using baseline-adjusted linear mixed-effects models, with propensity score weighting used for sensitivity analyses.

**Results:**

A total of 269 patients were included: 84 in the st-PNS group, 96 in the SON-PRF group, and 89 in the SON + SG-PRF group. NRS scores decreased over follow-up in all three groups. In the primary and both propensity score-weighted analyses, the overall NRS trajectory was lower with st-PNS than with SON-PRF, whereas no significant difference was observed between st-PNS and SON + SG-PRF. The overall GAD-7 trajectory was lower with SON + SG-PRF than with SON-PRF, and the overall PSQI trajectory was lower with SON + SG-PRF than with either st-PNS or SON-PRF; these differences remained statistically significant in both weighted analyses. The overall trajectories of depressive symptoms did not differ significantly among the groups. Exploratory analysis did not establish a difference in PHN risk among the three groups. No serious treatment-related adverse events occurred during follow-up.

**Conclusion:**

To our knowledge, this study is the first to compare multidimensional clinical outcomes of st-PNS, SON-PRF, and SON + SG-PRF for HZO-associated pain within the same cohort. st-PNS showed a relatively consistent analgesic benefit over SON-PRF, whereas SON + SG-PRF also provided analgesia and showed potential benefits for anxiety and sleep outcomes. These findings require confirmation in prospective studies.

## 1 Introduction

Herpes zoster ophthalmicus (HZO) is a distinct form of herpes zoster caused by reactivation of varicella-zoster virus in the trigeminal ganglion with involvement of the ophthalmic division of the trigeminal nerve. HZO accounts for approximately 8% of all herpes zoster cases, and its incidence is increasing (Fan et al., 2022; Niemeyer et al., 2024). In addition to periocular rash and pain, HZO may be complicated by conjunctivitis, uveitis, keratitis, and optic neuritis, with severe cases resulting in visual impairment (Ting et al., 2019). Zoster-associated pain may persist through the acute, subacute, and chronic phases of disease. Postherpetic neuralgia (PHN), one of its most common and difficult-to-manage complications, can adversely affect sleep, mood, and quality of life (Liesegang, 2008; Jiang et al., 2025). Previous research suggests that approximately 21% of patients with HZO develop PHN and that the risk may be further increased in those with ocular involvement such as keratitis or conjunctivitis (Wang et al., 2020; Warner et al., 2025). Older age, prodromal pain, and ocular inflammation may also increase the risk of progression to PHN (Borkar et al., 2013; Kawai et al., 2015). Effective management of HZO is therefore clinically important.

Short-term peripheral nerve stimulation (st-PNS) and supraorbital nerve pulsed radiofrequency (SON-PRF) are two neuromodulation techniques increasingly used to treat HZO-associated pain. st-PNS delivers continuous, adjustable electrical stimulation through an electrode positioned near the supraorbital and supratrochlear nerves, whereas SON-PRF uses a pulsed electric field to modulate the supraorbital nerve without causing destructive thermal injury (Chua et al., 2011). However, the initiation and persistence of HZO-associated pain involve not only abnormal excitability of peripheral sensory nerves but also sympathetic dysfunction and associated neuroinflammatory responses. Because SON-PRF primarily targets a peripheral sensory nerve, its influence on sympathetic activity may be limited.

Stellate ganglion pulsed radiofrequency (SG-PRF) has been used to treat facial and upper-limb PHN, but evidence regarding its use in HZO-associated pain remains lacking (Ding et al., 2019). In addition to its role in pain modulation, the stellate ganglion is an important component of the cervical sympathetic nervous system and may influence mood and sleep by regulating sympathetic tone and autonomic balance. Combining SG-PRF with SON-PRF may therefore provide additional clinical benefit by simultaneously modulating peripheral sensory and sympathetic pathways. Nevertheless, no study has directly compared SON-PRF with dual-target SON + SG-PRF, and the clinical outcomes of SON + SG-PRF relative to st-PNS remain unclear. We therefore conducted a retrospective cohort study to compare pain, emotional, sleep, and safety outcomes among st-PNS, SON-PRF, and SON + SG-PRF for HZO-associated pain.

## 2 Materials and methods

### 2.1 Study design

This single-center retrospective cohort study was reported in accordance with the Strengthening the Reporting of Observational Studies in Epidemiology (STROBE) guidelines for cohort studies. The study protocol was approved by the Medical Ethics Committee of China–Japan Friendship Hospital (approval no. 2023-KY-343). We retrospectively included patients with HZO who underwent st-PNS, SON-PRF, or SON + SG-PRF at China–Japan Friendship Hospital between January 2023 and January 2026. The study was conducted in accordance with the ethical principles of the Declaration of Helsinki.

### 2.2 Participants

The inclusion criteria were as follows: (1) a clinical diagnosis of HZO-associated pain distributed within the ophthalmic division (V1) of the trigeminal nerve; (2) a pretreatment Numerical Rating Scale (NRS) score ≥4; (3) receipt of st-PNS, SON-PRF, or SON + SG-PRF; (4) complete baseline data and follow-up outcome records required for this study; and (5) age ≥50 years.

The exclusion criteria were as follows: (1) pain involving the maxillary (V2) and/or mandibular (V3) divisions of the trigeminal nerve; (2) another cranial nerve or central nervous system disorder that could interfere with assessment of pain or neuropsychological outcomes, such as stroke or an intracranial space-occupying lesion; (3) coagulation dysfunction, infection at the puncture site, or a bleeding tendency; (4) severe cardiopulmonary dysfunction, malignancy, or a severe psychiatric disorder precluding outcome assessment; and (5) receipt during follow-up of a nerve block or another interventional procedure for HZO-associated pain outside the study protocol.

### 2.3 Treatment selection

Before treatment, clinicians fully explained the procedures, potential benefits, relevant risks, costs, and precautions associated with st-PNS, SON-PRF, and SON + SG-PRF. After receiving this information, patients independently selected their treatment; the investigators did not participate in treatment selection or alter the chosen intervention. Patients were classified into the st-PNS, SON-PRF, and SON + SG-PRF groups according to the treatment actually received.

### 2.4 st-PNS, SON-PRF, and SON+SG-PRF procedures

All procedures were performed by the same pain medicine team. st-PNS was performed under C-arm fluoroscopic guidance, whereas SON-PRF and SON + SG-PRF were performed under ultrasound guidance.

st-PNS: Patients were placed supine, underwent multiparameter physiological monitoring, and received intravenous access. The forehead, face, and neck were routinely disinfected and draped. After satisfactory local anesthesia, a puncture site approximately 1.5 cm superolateral to the eyebrow arch was selected, and the needle was advanced toward the anterior midline parallel to the supraorbital rim. After successful puncture, the stylet was removed and a stimulation lead (model 977A290 spinal cord stimulation lead) was inserted. The lead tip was advanced beyond the midline so that its eight contacts spanned the supraorbital and supratrochlear nerve territories. Continuous stimulation was delivered at 40 Hz with a pulse width of 750 μs. A current of approximately 3.8 mA was used as a reference and individualized within a range of approximately ± 1.0 mA according to the distribution of pain, paresthesia coverage, and patient tolerance. The target was comfortable paresthesia that covered the painful area and suppressed pain without causing intolerable discomfort. Frequency and pulse width remained unchanged throughout treatment, whereas current was adjusted according to clinical conditions. During the first 1–2 days, puncture-related tissue edema could increase the distance between the electrode and target nerves, requiring a modest increase in current in some patients. As the edema subsided, the current was reduced accordingly to maintain effective and comfortable stimulation coverage. After the introducer needle was removed, the lead was secured with sutures at and around the puncture site, and the incision was covered with a sterile dressing. The lead was removed after 7 days of continuous stimulation.

### 2.5 SON-PRF

Patients were placed supine and underwent multiparameter physiological monitoring. The forehead and face were routinely disinfected and draped. A puncture site approximately 1.5 cm superolateral to the eyebrow arch was selected. After satisfactory local anesthesia, a dedicated radiofrequency needle was advanced toward the anterior midline parallel to the supraorbital rim under ultrasound guidance. Sensory testing at 50 Hz and 0.3 V and motor testing at 2 Hz and 2.0 V were then performed. Once the stimulated area corresponded to the pain distribution, PRF was delivered at 42 °C, with a pulse width of 20 ms, a frequency of 2 Hz, and a duration of 600 s. The needle was then removed, and the puncture site was covered with a sterile dressing.

### 2.6 SG-PRF

Patients were placed supine and underwent multiparameter physiological monitoring. The neck was routinely disinfected and draped. Under ultrasound guidance, a dedicated radiofrequency needle was advanced in-plane after local anesthesia at the paravertebral region of C7 on the affected side. The needle tip was guided deep to the ipsilateral jugular vein and carotid artery until it reached the surface of the longus colli muscle adjacent to the stellate ganglion. Sensory testing at 50 Hz and 0.3 V and motor testing at 2 Hz and 2.0 V were then performed. Once the stimulated area corresponded to the pain distribution, PRF was delivered at 42 °C, with a pulse width of 20 ms, a frequency of 2 Hz, and a duration of 600 s. The needle was then removed, and the puncture site was covered with a sterile dressing. In the SON + SG-PRF group, SG-PRF was performed as described above after completion of SON-PRF.

### 2.7 Outcomes

Uniformly trained investigators extracted baseline characteristics and outcome data at weeks 1, 2, 4, 8, 12, and 24 after treatment from electronic medical records and follow-up records.

The primary outcomes were the longitudinal change in NRS score and the clinical response rate. Clinical response was defined as a ≥50% reduction in NRS score from baseline at follow-up. NRS scores range from 0 to 10, with higher scores indicating more severe pain. Secondary outcomes were the longitudinal changes in the 7-item Generalized Anxiety Disorder scale (GAD-7), 9-item Patient Health Questionnaire (PHQ-9), and Pittsburgh Sleep Quality Index (PSQI). These instruments assess anxiety symptoms, depressive symptoms, and sleep quality, respectively, with higher scores indicating greater severity. Safety outcomes included treatment-related adverse events occurring during treatment or follow-up.

The incidence of PHN was considered an exploratory outcome. PHN was evaluated only among patients whose disease duration was ≤90 days at treatment and was defined as pain with an NRS score ≥3 persisting in the original HZO-affected area at week 12 after treatment.

### 2.8 Statistical analysis

Statistical analyses were performed using R software (version 4.5.3). Normally distributed continuous variables are presented as mean ± standard deviation and were compared using one-way analysis of variance. Non-normally distributed variables are presented as median (interquartile range) and were compared using the Kruskal–Wallis H test. Categorical variables are presented as number and percentage [*n* (%)] and were compared using Pearson’s *χ*^2^ test.

Longitudinal changes in NRS, GAD-7, PHQ-9, and PSQI scores were analyzed using linear mixed-effects models that included treatment group, follow-up time, and their interaction as fixed effects and adjusted for the baseline value of the corresponding outcome. A random-intercept model, a random-intercept plus time random-slope model, and a random-intercept model with a first-order autoregressive residual correlation structure were compared using maximum likelihood estimation. The final model for each outcome was selected according to model convergence, the Akaike information criterion, and the Bayesian information criterion. The three pairwise comparisons of overall longitudinal profiles were adjusted using the Holm method. Because simultaneous evaluation of GAD-7, PHQ-9, and PSQI could increase the type I error rate, the overall group-related *p* values for these three secondary outcomes were additionally adjusted across outcomes using the Holm method. Single-time-point comparisons were considered exploratory, and the three pairwise comparisons were Holm-adjusted separately within each follow-up visit. Clinical response rates were first compared across the three groups at each follow-up visit using Pearson’s *χ*^2^ test, followed by Holm-adjusted post hoc pairwise comparisons.

To evaluate the robustness of the findings to measured treatment-selection confounding, generalized propensity scores for the three treatments were estimated using multinomial logistic regression including baseline covariates. Generalized overlap weighting was used as the primary propensity score sensitivity analysis, and stabilized inverse probability of treatment weighting (IPTW) was used as a supplementary sensitivity analysis. Covariate balance was assessed using the maximum absolute standardized difference across the three pairwise group comparisons, with an absolute standardized difference <0.10 considered acceptable. Weighted continuous outcomes and clinical response rates were analyzed using generalized estimating equations with robust standard errors and the same multiplicity-adjustment procedures as in the primary analysis. All tests were two-sided, and *p* < 0.05 was considered statistically significant.

## 3 Results

### 3.1 Patient selection

A total of 326 patients were screened. After excluding patients who did not meet the eligibility criteria, had incomplete follow-up data, or received another interventional treatment, 269 patients were included in the analysis: 84 in the st-PNS group, 96 in the SON-PRF group, and 89 in the SON + SG-PRF group. The patient selection and grouping process is shown in Figure 1.

**Figure 1:**
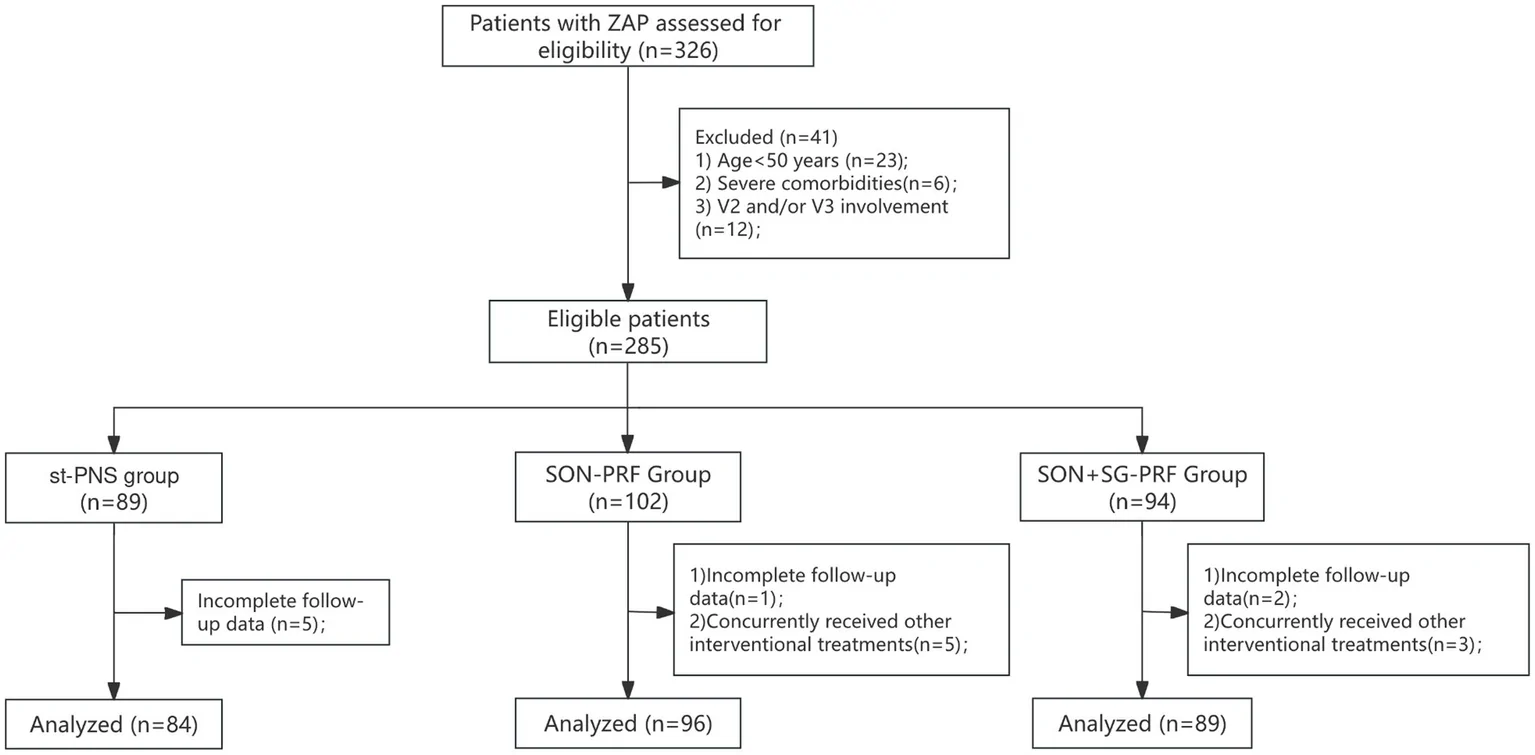
Flow diagram of participant selection.

### 3.2 Baseline characteristics

The study included 269 patients: 84 in the st-PNS group, 96 in the SON-PRF group, and 89 in the SON + SG-PRF group. Age, sex, affected side, disease duration, history of antiviral therapy, and baseline NRS, GAD-7, PHQ-9, and PSQI scores are presented in Table 1.

**Table 1:** Baseline characteristics of the study groups.

Characteristic	st-PNS group (*n* = 84)	SON-PRF group (*n* = 96)	SON+SG-PRF group (*n* = 89)	*p* value
Age (years)	67.39 ± 8.49	67.38 ± 10.35	68.64 ± 10.14	0.609
Sex				0.183
Male	44 (52.4%)	59 (61.5%)	43 (48.3%)	
Female	40 (47.6%)	37 (38.5%)	46 (51.7%)	
Affected side				0.123
Left	40 (47.6%)	60 (62.5%)	47 (52.8%)	
Right	44 (52.4%)	36 (37.5%)	42 (47.2%)	
Disease duration				0.800
<1 month	24 (28.6%)	27 (28.1%)	29 (32.6%)	
1–3 months	52 (61.9%)	59 (61.5%)	48 (53.9%)	
>3 months	8 (9.5%)	10 (10.4%)	12 (13.5%)	
History of antiviral therapy				0.287
Yes	63 (75.0%)	65 (67.7%)	57 (64.0%)	
No	21 (25.0%)	31 (32.3%)	32 (36.0%)	
Baseline NRS score	6.27 ± 1.39	6.55 ± 1.33	6.46 ± 1.38	0.387
Baseline GAD-7 score	11.77 ± 2.69	11.54 ± 2.60	11.58 ± 2.91	0.837
Baseline PHQ-9 score	10.40 ± 2.89	10.54 ± 2.80	10.66 ± 2.78	0.835
Baseline PSQI score	13.95 ± 2.14	14.34 ± 2.16	14.07 ± 2.07	0.442

### 3.3 Changes in pain-related outcomes

Comparison of candidate models showed that the random-intercept plus time random-slope model provided a better fit to the NRS data than either the random-intercept model or the random-intercept model with a first-order autoregressive residual correlation structure; it was therefore selected for the final analysis (Supplementary Table S1).

NRS scores decreased over follow-up in all three groups (Figure 2A). The baseline-adjusted linear mixed-effects model showed significant overall time-related and joint group effects (*χ*^2^ = 175.649, df = 15, *p* < 0.001; and *χ*^2^ = 28.292, df = 12, *p* = 0.005, respectively), whereas the group-by-time interaction was not significant (*χ*^2^ = 14.387, df = 10, *p* = 0.156; Table 2). Pairwise comparisons of the overall profiles showed lower NRS scores with st-PNS and SON + SG-PRF than with SON-PRF (Holm-adjusted *p* = 0.005 and *p* = 0.028, respectively), whereas st-PNS and SON + SG-PRF did not differ significantly (Holm-adjusted *p* = 0.551). Exploratory single-time-point comparisons showed lower NRS scores with st-PNS than with SON-PRF at weeks 2, 4, 8, 12, and 24, and lower scores with SON + SG-PRF than with SON-PRF at weeks 2 and 4 (Supplementary Table S2).

**Figure 2:**
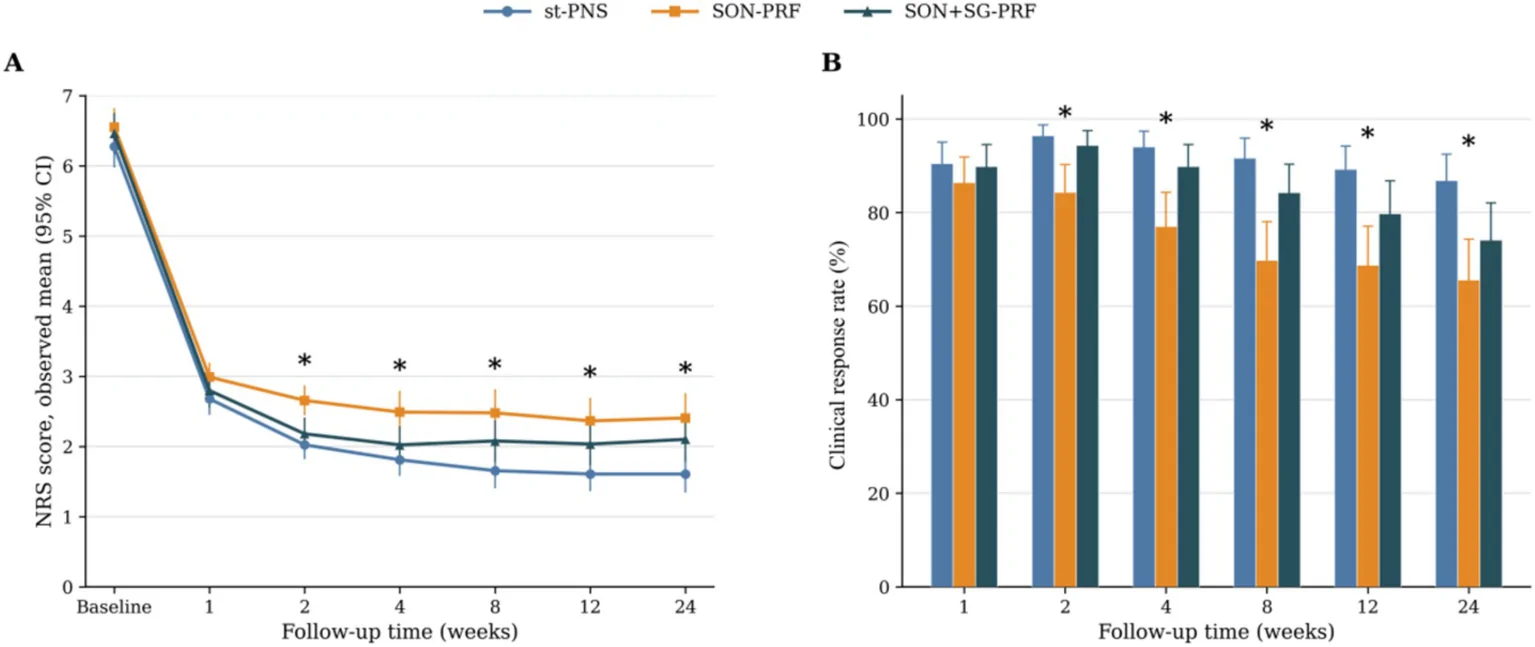
Changes in pain outcomes during the 24-week follow-up. **(A)** Observed mean NRS scores with 95% confidence intervals. **(B)** Clinical response rates with 95% confidence intervals.Asterisks indicate exploratory overall three-group differences at individual follow-up visits after Holm adjustment across the six visits (*adjusted *p* < 0.05). Post hoc pairwise comparisons were Holm-adjusted separately within each follow-up visit.

**Table 2:** Overall model tests and pairwise comparisons of NRS profiles from the linear mixed-effects model.

Effect or comparison	*χ* ^2^	Df	*p* value	Holm-adjusted *p* value
Overall model tests				
Overall time-related effect	175.649	15	<0.001	–
Group × time	14.387	10	0.156	–
Joint group effect	28.292	12	0.005	–
Pairwise profile comparisons				
st-PNS vs SON-PRF	21.154	6	0.002	0.005
st-PNS vs SON+SG-PRF	4.947	6	0.551	0.551
SON-PRF vs SON+SG-PRF	15.942	6	0.014	0.028

The overall clinical response rates did not differ among the three groups at week 1 (*p* = 0.646), but differed significantly at weeks 2, 4, 8, 12, and 24 (all *p* ≤ 0.007; Figure 2B). Post hoc pairwise comparisons showed higher response rates with st-PNS than with SON-PRF at weeks 2, 4, 8, 12, and 24 (Holm-adjusted *p* = 0.021, 0.004, <0.001, 0.003, and 0.003, respectively). No significant differences were observed between st-PNS and SON + SG-PRF at any follow-up visit. Response rates were higher with SON + SG-PRF than with SON-PRF at weeks 4 and 8 (Holm-adjusted *p* = 0.040 at both visits), but not at weeks 2, 12, or 24 (Supplementray Table S3).

### 3.4 Changes in anxiety and depression scores

Comparison of candidate models showed that the random-intercept plus time random-slope model provided a better fit to the GAD-7 and PHQ-9 data than either the random-intercept model or the random-intercept model with a first-order autoregressive residual correlation structure; it was therefore selected for the final analyses (Supplementary Table S1).

GAD-7 scores decreased over follow-up in all three groups (Figure 3A). The baseline-adjusted linear mixed-effects model showed significant overall time-related, group-by-time interaction, and joint group effects (*χ*^2^ = 242.958, df = 15, *p* < 0.001; *χ*^2^ = 21.815, df = 10, *p* = 0.016; and *χ*^2^ = 29.784, df = 12, *p* = 0.003, respectively; Supplementary Table S4). Pairwise comparisons of the overall profiles showed lower GAD-7 scores with SON + SG-PRF and st-PNS than with SON-PRF (Holm-adjusted *p* < 0.001 and *p* = 0.049, respectively), whereas st-PNS and SON + SG-PRF did not differ significantly (Holm-adjusted *p* = 0.774). Exploratory single-time-point comparisons are presented in Supplementary Table S5.

**Figure 3:**
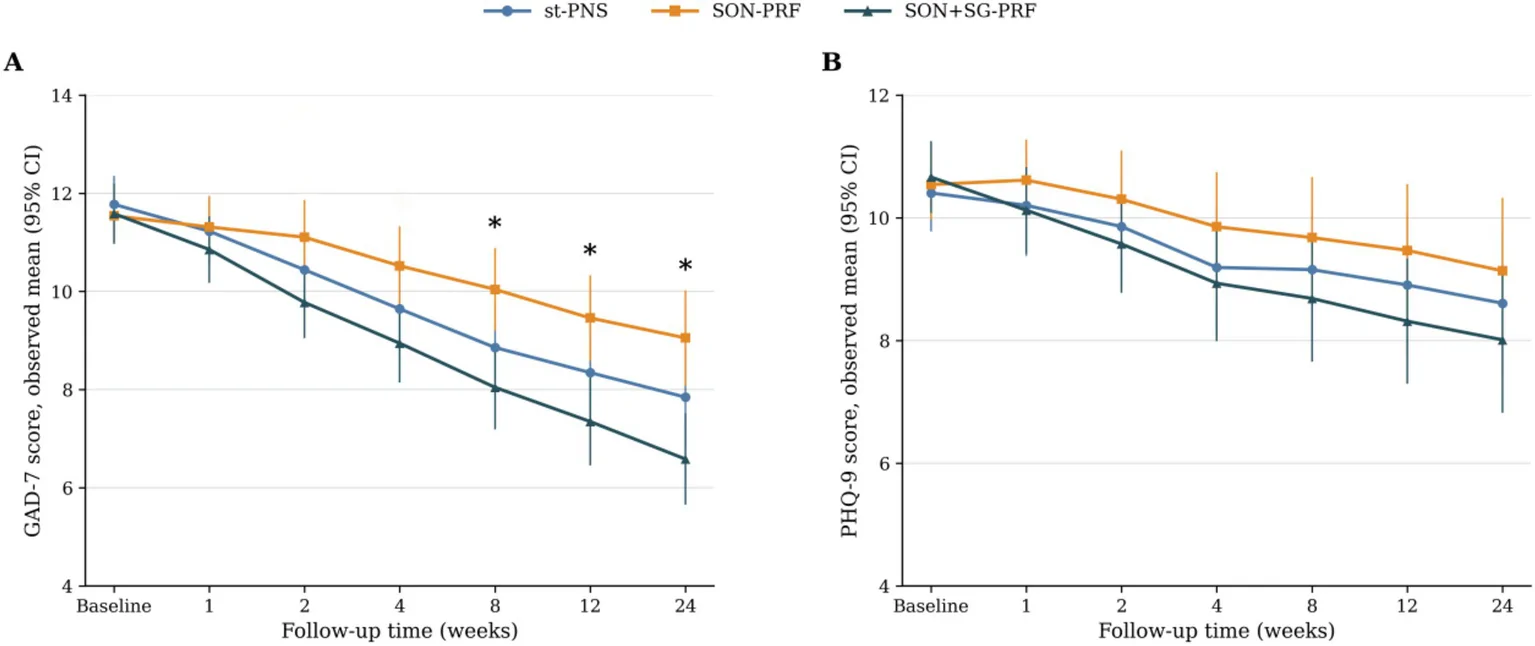
Longitudinal changes in anxiety and depressive symptom scores during the 24-week follow-up. **(A)** Observed mean GAD-7 scores. **(B)** Observed mean PHQ-9 scores. Asterisks indicate exploratory overall between-group differences at individual follow-up visits after Holm correction across the six visits (*adjusted *p* < 0.05). Longitudinal inference was based on baseline-adjusted linear mixed-effects models.

PHQ-9 scores also decreased over time in all three groups (Figure 3B). The overall time-related effect was significant (*χ*^2^ = 52.071, df = 15, *p* < 0.001), whereas neither the group-by-time interaction nor the joint group effect was significant (*χ*^2^ = 2.276, df = 10, *p* = 0.994; and *χ*^2^ = 5.931, df = 12, *p* = 0.920, respectively). None of the three pairwise comparisons of overall profiles was significant (Supplementary Tables S6 and S7).

Because simultaneous evaluation of GAD-7, PHQ-9, and PSQI could increase the type I error rate, the overall group-related *p* values for these secondary outcomes were additionally Holm-adjusted across outcomes. After adjustment, the overall group difference remained significant for GAD-7 (adjusted *p* = 0.006; Supplementray Table S8) and remained nonsignificant for PHQ-9 (adjusted *p* = 0.920; Supplementary Table S8).

### 3.5 Changes in sleep quality

Comparison of candidate models showed that the random-intercept model provided a better fit to the PSQI data than either the random-intercept plus time random-slope model or the random-intercept model with a first-order autoregressive residual correlation structure; it was therefore selected for the final analysis (Supplementray Table S1).

PSQI scores decreased over follow-up in all three groups (Figure 4). The baseline-adjusted linear mixed-effects model showed significant overall time-related and joint group effects (*χ*^2^ = 318.701, df = 15, *p* < 0.001; and *χ*^2^ = 99.742, df = 12, *p* < 0.001, respectively), whereas the group-by-time interaction was not significant (*χ*^2^ = 10.160, df = 10, *p* = 0.427). Pairwise comparisons of overall profiles showed lower PSQI scores with SON + SG-PRF than with st-PNS and SON-PRF (Holm-adjusted *p* = 0.004 and *p* < 0.001, respectively); scores were also lower with st-PNS than with SON-PRF (Holm-adjusted *p* < 0.001; Supplementray Table S9). Exploratory single-time-point comparisons are presented in Supplementray Table S10.

**Figure 4:**
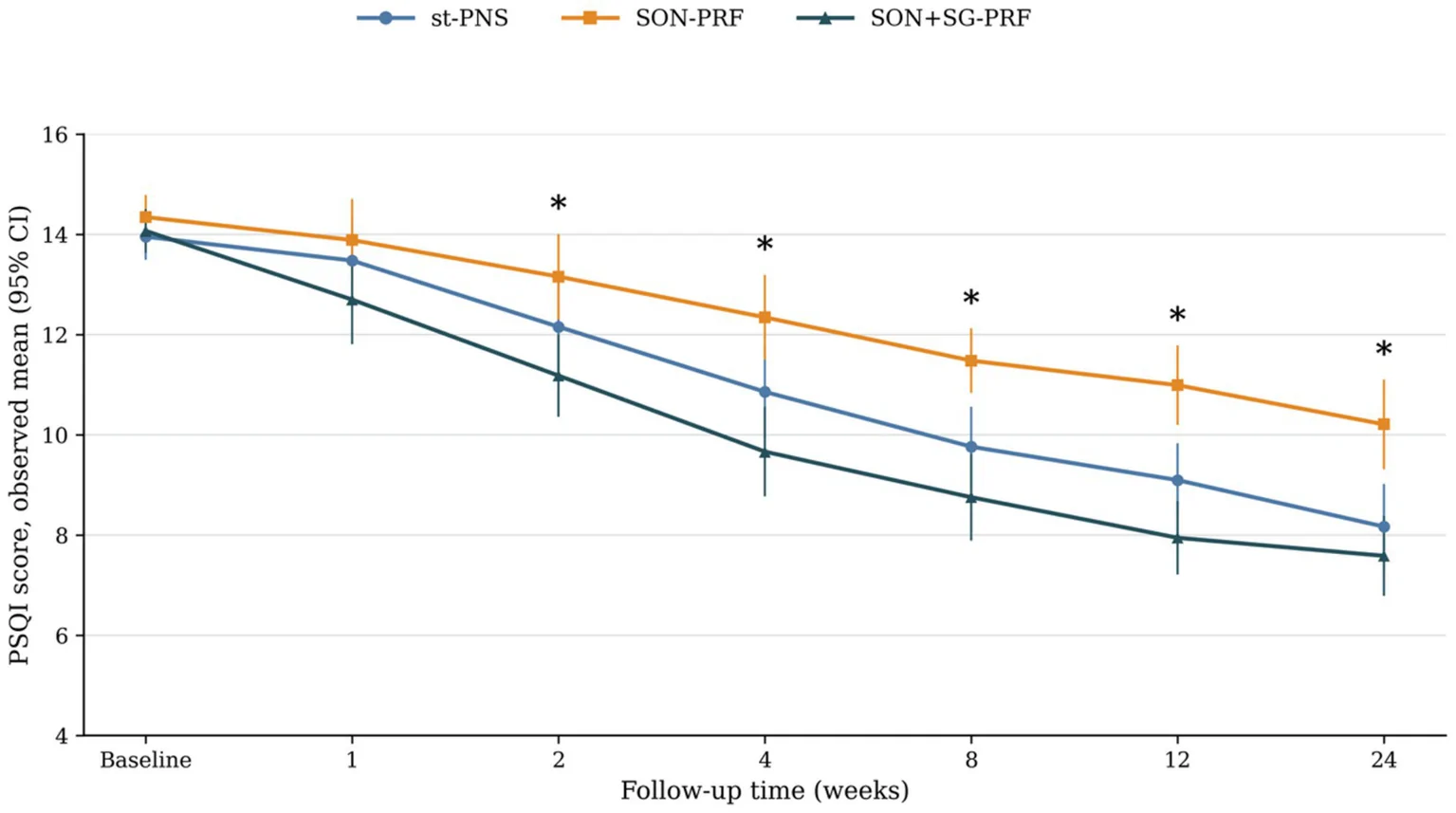
Changes in PSQI scores during the 24-week follow-up. Asterisks indicate exploratory overall between-group differences at individual follow-up visits after Holm correction across the six visits (*adjusted *p* < 0.05). Longitudinal inference was based on the baseline-adjusted linear mixed-effects model.

After Holm adjustment across the overall group-related *p* values for GAD-7, PHQ-9, and PSQI, the overall group difference for PSQI remained significant (adjusted *p* < 0.001; Supplementray Table S8).

### 3.6 Incidence of PHN

Among the 239 patients with a disease duration ≤90 days at treatment, PHN occurred in 9 of 76 patients (11.8%) in the st-PNS group, 20 of 86 (23.3%) in the SON-PRF group, and 13 of 77 (16.9%) in the SON + SG-PRF group. The overall three-group comparison was not statistically significant (*χ*^2^ = 3.666, df = 2, *p* = 0.160; Figure 5). Exploratory pairwise comparisons yielded a risk difference of −11.4 percentage points for st-PNS versus SON-PRF (95% CI, −22.8 to 0.5), −5.0 percentage points for st-PNS versus SON + SG-PRF (95% CI, −16.4 to 6.3), and 6.4 percentage points for SON-PRF versus SON + SG-PRF (95% CI, −6.1 to 18.4). Although PHN incidence differed numerically among the groups, all 95% confidence intervals for the risk differences included zero and were wide. These findings therefore did not establish a difference in PHN risk among the three treatment strategies.

**Figure 5:**
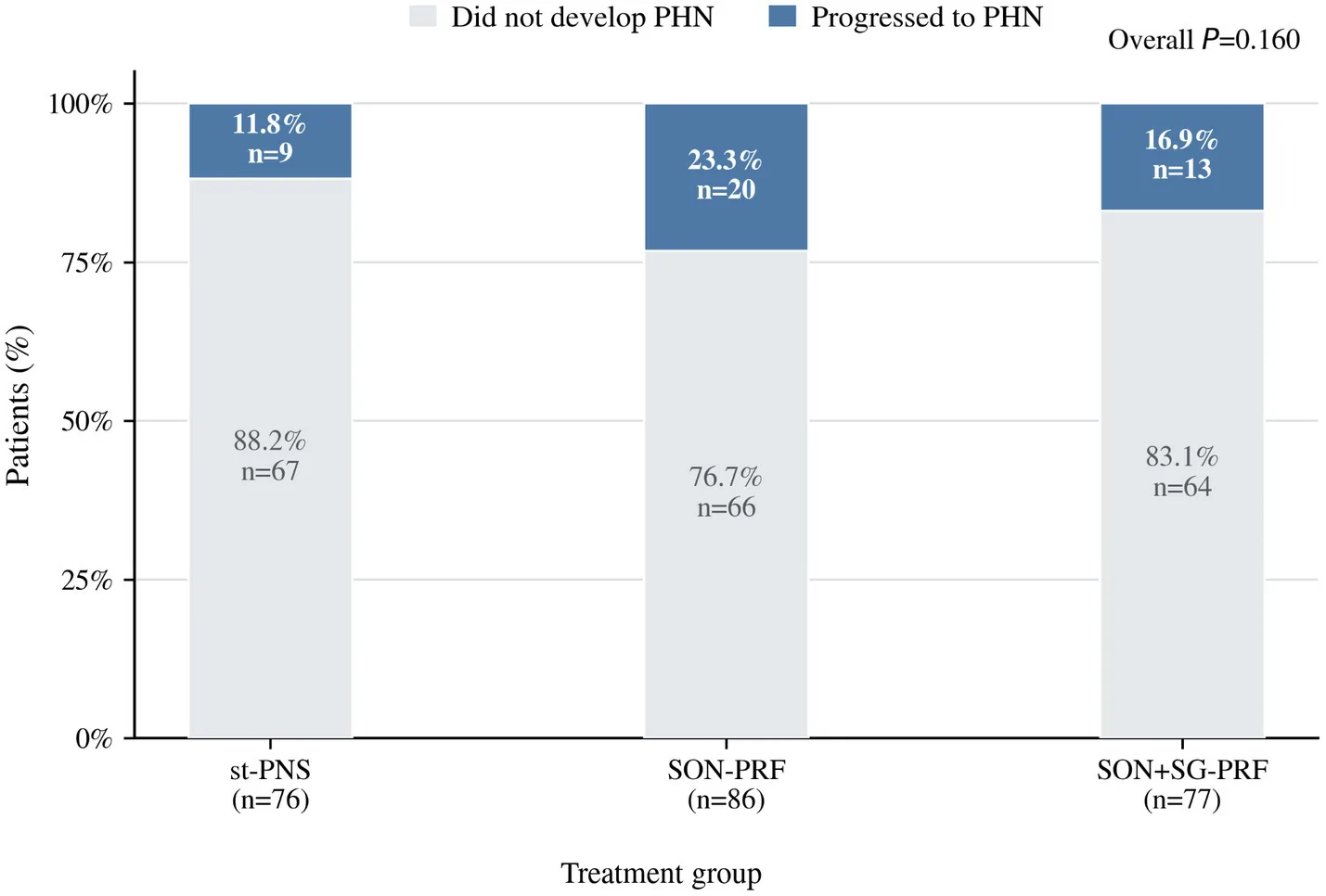
Progression to postherpetic neuralgia across the three treatment groups.

### 3.7 Propensity score-weighted sensitivity analyses

Both generalized overlap weighting and stabilized IPTW retained all 269 patients. The effective sample sizes were 250.0 and 249.6, respectively. Stabilized IPTW weights ranged from 0.59 to 2.19, with no evidence of extreme weights. The maximum absolute standardized difference across pairwise group comparisons was 0.311 before weighting and decreased to 0.074 after generalized overlap weighting and 0.086 after stabilized IPTW. All baseline covariates included in the propensity score model met the prespecified balance criterion after weighting (Supplementray Figure S1 and Table S11).

Weighted pain outcomes were generally consistent with the primary analysis. The overall group-related effect for NRS was significant with both generalized overlap weighting and stabilized IPTW (*p* = 0.010 and *p* = 0.009, respectively). In both weighted analyses, the overall NRS trajectory differed significantly between st-PNS and SON-PRF (Holm-adjusted *p* = 0.038 and *p* = 0.035, respectively), but not between st-PNS and SON + SG-PRF or between SON-PRF and SON + SG-PRF. For clinical response rates, both weighting methods showed higher rates with st-PNS than with SON-PRF at weeks 2, 4, 8, 12, and 24, whereas st-PNS and SON + SG-PRF did not differ at any visit. Differences between SON + SG-PRF and SON-PRF were observed mainly at weeks 2, 4, and 8 (Supplementray Tables S12–S14).

For anxiety, the overall group-related effect for GAD-7 remained significant after Holm adjustment across the three secondary outcomes in both the generalized overlap-weighted and stabilized IPTW analyses (adjusted *p* = 0.014 and *p* = 0.011, respectively). Under both weighting methods, only the overall trajectory comparison between SON + SG-PRF and SON-PRF was significant; the other two pairwise comparisons were not. PHQ-9 showed no overall group-related difference in either weighted analysis (adjusted *p* = 0.890 and *p* = 0.880, respectively; Supplementary Tables S8, S12, and S13).

For sleep quality, the overall group-related difference in PSQI remained significant after Holm adjustment across outcomes in both weighted analyses (both *p* < 0.001). After generalized overlap weighting, the overall trajectories differed significantly between st-PNS and SON-PRF, between st-PNS and SON + SG-PRF, and between SON-PRF and SON + SG-PRF (Holm-adjusted *p* < 0.001, *p* =0.006, and *p* < 0.001, respectively). Stabilized IPTW yielded consistent results (*p* < 0.001, p =0.011, and p < 0.001, respectively; Supplementary Tables S8, S12, and S13).

### 3.8 Adverse events

No serious treatment-related adverse events occurred in any group. One patient in the st-PNS group developed a local hematoma at the puncture site, and one patient in the SON-PRF group developed upper-eyelid swelling; both events were mild.

## 4 Discussion

To our knowledge, this study is the first to directly compare clinical outcomes of st-PNS, SON-PRF, and SON + SG-PRF for HZO-associated pain. The overall NRS trajectory was lower with st-PNS than with SON-PRF, and clinical response rates were higher with st-PNS from weeks 2 through 24. These pain-related findings remained consistent in both propensity score-weighted analyses, suggesting that st-PNS may provide more sustained analgesia. The overall NRS trajectory did not differ significantly between SON + SG-PRF and st-PNS, whereas the advantage of SON + SG-PRF over SON-PRF was observed only in the primary analysis. The overall GAD-7 trajectory was lower with SON + SG-PRF than with SON-PRF, and the overall PSQI trajectory was lower with SON + SG-PRF than with either st-PNS or SON-PRF; these findings were consistent in both weighted analyses. The overall trajectory of depressive symptoms did not differ among the groups. Collectively, the principal benefit of st-PNS was sustained analgesia, whereas SON + SG-PRF was associated with favorable anxiety and sleep outcomes in addition to pain relief.

The overall NRS trajectory was lower with st-PNS than with SON-PRF, and this finding remained consistent in both propensity score-weighted analyses. Holm-adjusted exploratory post hoc comparisons further showed higher clinical response rates with st-PNS than with SON-PRF at weeks 12 and 24 (89.3% vs. 68.8% and 86.9% vs. 65.6%, respectively), suggesting that st-PNS may provide relatively sustained analgesia. Previous research suggests that st-PNS continuously recruits large myelinated Aβ fibers, engages inhibitory interneurons in the substantia gelatinosa of the dorsal horn, and thereby reduces synaptic transmission of nociceptive Aδ/C-fiber signals to second-order projection neurons. It may also engage local inflammatory pathways, the autonomic nervous system, and endogenous pain-inhibitory pathways, producing both peripheral and central analgesic effects (Kaye et al., 2021; Lin et al., 2020). By contrast, SON-PRF may alleviate neuropathic pain by suppressing excitatory neurotransmitter release, modulating pain-related gene expression, and increasing neurotrophic factors such as glial cell line-derived neurotrophic factor (Sluijter et al., 2010; Vallejo et al., 2013; Lin et al., 2025). The two treatments also differed in stimulation duration and anatomical coverage. st-PNS was delivered over a longer period and allowed dynamic adjustment of current. Previous research suggests that sustained stimulation may repeatedly reinforce gate-control mechanisms and promote activity-dependent synaptic plasticity (Ong Sio et al., 2023). Frontal HZO pain often involves terminal V1 branches, including the supraorbital and supratrochlear nerves. The linear st-PNS lead can cover both territories, whereas SON-PRF targets the supraorbital nerve alone. If the supratrochlear nerve or adjacent V1 branches also contribute to pain, single-target modulation may provide incomplete coverage. Together, these factors may help explain the more consistent analgesic effect observed with st-PNS than with SON-PRF.

HZO-associated pain is frequently accompanied by anxiety and sleep disturbance. Anxiety, disrupted sleep, and sustained hyperarousal may in turn amplify pain perception, creating a mutually reinforcing cycle (Du et al., 2021). In this study, the overall GAD-7 trajectory was lower with SON + SG-PRF than with SON-PRF, and the overall PSQI trajectory was lower with SON + SG-PRF than with either st-PNS or SON-PRF. These findings may reflect both pain relief and coordinated modulation of sensory and sympathetic pathways. Previous studies have shown that after nerve injury, sympathetic fibers may sprout abnormally within sensory ganglia; norepinephrine released from their terminals can increase sensory-neuron excitability and induce abnormal synchronous firing in adjacent neurons, contributing to sympathetically maintained spontaneous pain and hyperalgesia (McLachlan et al., 1993; Luo et al., 2022; Zheng et al., 2022). Stellate ganglion interventions may reduce this amplification process by decreasing sympathetic excitability and norepinephrine release. They may also relieve sympathetic vasoconstriction, improve local perfusion and the metabolic environment of neural tissue, and thereby attenuate neuroinflammation and pain sensitization (Wang et al., 2005; Luo et al., 2022). In the present study, improvements in anxiety and sleep outcomes may have initially resulted from reduced pain-related emotional burden and fewer nocturnal awakenings. Animal studies further suggest that stellate ganglion block may modulate central stress and arousal states related to anxiety by influencing chronic stress responses and the noradrenergic locus coeruleus–basolateral amygdala pathway (Wang et al., 2017; Wang et al., 2025). Clinical studies have also reported reductions in norepinephrine and cortisol levels after stellate ganglion block, accompanied by improvements in sleep duration, sleep efficiency, and N3 sleep (Gu et al., 2022; Liu et al., 2025). These mechanistic interpretations are based on studies of stellate ganglion block and animal models. Large prospective studies of SG-PRF incorporating autonomic, neuroendocrine, and inflammatory biomarkers are needed to evaluate them further.

Persistent nociceptive input caused by varicella-zoster virus reactivation may increase neuronal excitability, activate glial cells, and remodel pain-related neural networks, thereby promoting central sensitization (Liu et al., 2024; Yan X. et al., 2026). Reducing abnormal sensory input during the acute or subacute phase may therefore help lower the risk of pain chronification (Yan Y. et al., 2026). However, the present exploratory analysis did not establish a difference in PHN risk among the three treatment strategies, and the confidence intervals around the risk estimates were wide. The observed numerical differences therefore cannot be interpreted as evidence of a preventive effect. Larger prospective studies are needed to evaluate how different neuromodulation strategies affect the risk of PHN. Regarding safety, no serious treatment-related adverse events occurred during follow-up, and only two mild events were observed.

This study has several limitations. First, it was a single-center retrospective study in which patients selected their treatment. Treatment choice may have been influenced by disease severity, socioeconomic circumstances, and other factors. Although propensity score weighting was used to adjust for measured baseline characteristics, residual confounding by unmeasured factors and treatment-selection bias cannot be excluded. Second, the three treatment strategies differed in stimulation modality, treatment duration, and anatomical coverage. The observed between-group differences therefore reflect the overall effects of these treatment strategies and cannot isolate the respective contributions of stimulation modality, treatment duration, and target coverage. Finally, autonomic function and neuroendocrine and inflammatory biomarkers were not measured, precluding evaluation of the mechanisms through which SG-PRF might affect pain, anxiety, and sleep.

## 5 Conclusion

This study directly compared clinical outcomes of st-PNS, SON-PRF, and SON + SG-PRF for HZO-associated pain. st-PNS showed a relatively consistent and sustained analgesic benefit, whereas SON + SG-PRF also provided analgesia and showed potential benefits for anxiety and sleep outcomes. No serious treatment-related adverse events occurred in any group during follow-up. Given the retrospective design and differences among the treatment strategies, these findings require confirmation in large, multicenter, prospective randomized controlled trials.

## Data Availability

The original contributions presented in the study are included in the article/Supplementary material, further inquiries can be directed to the corresponding author.
