# Supplementary material for: Comparison of short-term peripheral nerve stimulation, single-target supraorbital nerve pulsed radiofrequency, and dual-target supraorbital nerve–stellate ganglion pulsed radiofrequency for herpes zoster ophthalmicus–associated pain: a retrospective cohort study
Source: Front Neurosci. 2026 Oct 23;20:1976324. doi: 10.3389/fnins.2026.1976324 (PMC13739791; doi:10.3389/fnins.2026.1976324)
Supplement: Supplementary file 1 [file Data_sheet_1.docx]

**Table S1. Comparison of random-effect and within-participant correlation structures for longitudinal outcomes**

| **Outcome** | **Model structure** | **AIC** | **BIC** | **Selection** |
| --- | --- | --- | --- | --- |
| NRS | Random intercept | 4362.5 | 4475.6 |  |
|  | Random intercept + time random slope | 4026.4 | 4150.3 | Selected |
|  | Random intercept + AR(1) | 4078.0 | 4196.5 |  |
| GAD-7 | Random intercept | 7023.6 | 7136.7 |  |
|  | Random intercept + time random slope | 6167.1 | 6291.0 | Selected |
|  | Random intercept + AR(1) | 6216.8 | 6335.3 |  |
| PHQ-9 | Random intercept | 7693.0 | 7806.1 |  |
|  | Random intercept + time random slope | 7051.8 | 7175.7 | Selected |
|  | Random intercept + AR(1) | 7220.4 | 7338.9 |  |
| PSQI | Random intercept | 8853.0 | 8966.1 | Selected |
|  | Random intercept + time random slope | 8856.5 | 8980.4 |  |
|  | Random intercept + AR(1) | 8855.5 | 8974.0 |  |

AIC, Akaike information criterion; AR(1), first-order autoregressive; BIC, Bayesian information criterion.

**Table S2. Time-specific pairwise comparisons of NRS scores from the linear mixed-effects model.**

| **Time** | **Comparison** | **Adjusted mean difference** | **SE** | **95% CI** | **Raw P** | **Holm-adjusted P** |
| --- | --- | --- | --- | --- | --- | --- |
| Week 1 | st-PNS vs SON-PRF | −0.169 | 0.125 | −0.415 to 0.078 | 0.179 | 0.538 |
| Week 1 | st-PNS vs SON+SG-PRF | −0.024 | 0.127 | −0.274 to 0.227 | 0.853 | 0.853 |
| Week 1 | SON-PRF vs SON+SG-PRF | 0.145 | 0.123 | −0.097 to 0.387 | 0.240 | 0.538 |
| Week 2 | st-PNS vs SON-PRF | −0.490 | 0.134 | −0.755 to −0.225 | <0.001 | 0.001 |
| Week 2 | st-PNS vs SON+SG-PRF | −0.060 | 0.137 | −0.330 to 0.209 | 0.659 | 0.659 |
| Week 2 | SON-PRF vs SON+SG-PRF | 0.430 | 0.132 | 0.170 to 0.690 | 0.001 | 0.003 |
| Week 4 | st-PNS vs SON-PRF | −0.538 | 0.152 | −0.836 to −0.239 | <0.001 | 0.001 |
| Week 4 | st-PNS vs SON+SG-PRF | −0.117 | 0.154 | −0.421 to 0.186 | 0.447 | 0.447 |
| Week 4 | SON-PRF vs SON+SG-PRF | 0.420 | 0.149 | 0.127 to 0.714 | 0.005 | 0.010 |
| Week 8 | st-PNS vs SON-PRF | −0.682 | 0.174 | −1.025 to −0.339 | <0.001 | <0.001 |
| Week 8 | st-PNS vs SON+SG-PRF | −0.328 | 0.177 | −0.677 to 0.021 | 0.065 | 0.080 |
| Week 8 | SON-PRF vs SON+SG-PRF | 0.354 | 0.171 | 0.016 to 0.691 | 0.040 | 0.080 |
| Week 12 | st-PNS vs SON-PRF | −0.615 | 0.201 | −1.010 to −0.220 | 0.002 | 0.007 |
| Week 12 | st-PNS vs SON+SG-PRF | −0.331 | 0.204 | −0.733 to 0.071 | 0.106 | 0.213 |
| Week 12 | SON-PRF vs SON+SG-PRF | 0.284 | 0.198 | −0.105 to 0.673 | 0.152 | 0.213 |
| Week 24 | st-PNS vs SON-PRF | −0.657 | 0.230 | −1.109 to −0.205 | 0.005 | 0.014 |
| Week 24 | st-PNS vs SON+SG-PRF | −0.398 | 0.234 | −0.859 to 0.062 | 0.089 | 0.179 |
| Week 24 | SON-PRF vs SON+SG-PRF | 0.258 | 0.226 | −0.187 to 0.703 | 0.254 | 0.254 |

Note. Pairwise comparisons were adjusted using the Holm method within each follow-up visit. No additional adjustment was applied across follow-up visits; these time-point-specific comparisons were considered exploratory.

**Table S3. Clinical response rates and post-hoc pairwise comparisons at each follow-up visit.**

**Panel A. Clinical response rates and overall three-group comparisons**

| **Time** | **st-PNS** | **SON-PRF** | **SON+SG-PRF** | **Overall P** |
| --- | --- | --- | --- | --- |
| Week 1 | 76/84 (90.5%) | 83/96 (86.5%) | 80/89 (89.9%) | 0.646 |
| Week 2 | 81/84 (96.4%) | 81/96 (84.4%) | 84/89 (94.4%) | 0.007 |
| Week 4 | 79/84 (94.0%) | 74/96 (77.1%) | 80/89 (89.9%) | 0.002 |
| Week 8 | 77/84 (91.7%) | 67/96 (69.8%) | 75/89 (84.3%) | <0.001 |
| Week 12 | 75/84 (89.3%) | 66/96 (68.8%) | 71/89 (79.8%) | 0.003 |
| Week 24 | 73/84 (86.9%) | 63/96 (65.6%) | 66/89 (74.2%) | 0.004 |

**Panel B. Post-hoc pairwise comparisons**

| **Time** | **Comparison** | **Raw P** | **Holm-adjusted P** |
| --- | --- | --- | --- |
| Week 1 | st-PNS vs SON-PRF | 0.402 | 1.000 |
| Week 1 | st-PNS vs SON+SG-PRF | 0.897 | 1.000 |
| Week 1 | SON-PRF vs SON+SG-PRF | 0.472 | 1.000 |
| Week 2 | st-PNS vs SON-PRF | 0.007 | 0.021 |
| Week 2 | st-PNS vs SON+SG-PRF | 0.522 | 0.522 |
| Week 2 | SON-PRF vs SON+SG-PRF | 0.029 | 0.057 |
| Week 4 | st-PNS vs SON-PRF | 0.001 | 0.004 |
| Week 4 | st-PNS vs SON+SG-PRF | 0.316 | 0.316 |
| Week 4 | SON-PRF vs SON+SG-PRF | 0.020 | 0.040 |
| Week 8 | st-PNS vs SON-PRF | <0.001 | <0.001 |
| Week 8 | st-PNS vs SON+SG-PRF | 0.136 | 0.136 |
| Week 8 | SON-PRF vs SON+SG-PRF | 0.020 | 0.040 |
| Week 12 | st-PNS vs SON-PRF | <0.001 | 0.003 |
| Week 12 | st-PNS vs SON+SG-PRF | 0.085 | 0.170 |
| Week 12 | SON-PRF vs SON+SG-PRF | 0.087 | 0.170 |
| Week 24 | st-PNS vs SON-PRF | <0.001 | 0.003 |
| Week 24 | st-PNS vs SON+SG-PRF | 0.035 | 0.070 |
| Week 24 | SON-PRF vs SON+SG-PRF | 0.207 | 0.207 |

Pairwise comparisons were adjusted using the Holm method within each follow-up visit. No additional adjustment was applied across follow-up visits; these time-point-specific comparisons were considered exploratory.

**Table S4. Overall model tests and pairwise comparisons of GAD-7 trajectories using the linear mixed-effects model.**

| **Effect or comparison** | **χ²** | **df** | **P value** | **Adjusted P value** |
| --- | --- | --- | --- | --- |
| **Overall model tests** |  |  |  |  |
| Overall time-related effect | 242.958 | 15 | <0.001 | — |
| Group × time | 21.815 | 10 | 0.016 | — |
| Joint group effect | 29.784 | 12 | 0.003 | 0.006 |
| **Pairwise profile comparisons** |  |  |  |  |
| st-PNS vs SON-PRF | 14.463 | 6 | 0.025 | 0.049 |
| st-PNS vs SON+SG-PRF | 3.270 | 6 | 0.774 | 0.774 |
| SON-PRF vs SON+SG-PRF | 26.174 | 6 | <0.001 | <0.001 |

The model included participant-specific random intercepts and time random slopes and was adjusted for baseline GAD-7. For the joint group effect, the adjusted P value was obtained by applying the Holm procedure across the three secondary outcomes (GAD-7, PHQ-9, and PSQI). For pairwise profile comparisons, Holm adjustment was applied to the three between-group comparisons within GAD-7. GAD-7, 7-item Generalized Anxiety Disorder scale; PSQI, Pittsburgh Sleep Quality Index.

**Table S5. Time-specific pairwise comparisons of GAD-7 scores from the linear mixed-effects model.**

| **Time** | **Comparison** | **Adjusted mean difference** | **SE** | **95% CI** | **Raw P** | **Holm-adjusted P** |
| --- | --- | --- | --- | --- | --- | --- |
| Week 1 | st-PNS vs SON-PRF | −0.329 | 0.297 | −0.913 to 0.255 | 0.269 | 0.537 |
| Week 1 | st-PNS vs SON+SG-PRF | 0.174 | 0.302 | −0.420 to 0.769 | 0.564 | 0.564 |
| Week 1 | SON-PRF vs SON+SG-PRF | 0.503 | 0.292 | −0.072 to 1.078 | 0.086 | 0.258 |
| Week 2 | st-PNS vs SON-PRF | −0.906 | 0.332 | −1.559 to −0.253 | 0.007 | 0.014 |
| Week 2 | st-PNS vs SON+SG-PRF | 0.467 | 0.338 | −0.198 to 1.132 | 0.168 | 0.168 |
| Week 2 | SON-PRF vs SON+SG-PRF | 1.373 | 0.327 | 0.730 to 2.017 | <0.001 | <0.001 |
| Week 4 | st-PNS vs SON-PRF | −1.120 | 0.393 | −1.893 to −0.347 | 0.005 | 0.009 |
| Week 4 | st-PNS vs SON+SG-PRF | 0.501 | 0.400 | −0.286 to 1.288 | 0.211 | 0.211 |
| Week 4 | SON-PRF vs SON+SG-PRF | 1.621 | 0.387 | 0.860 to 2.383 | <0.001 | <0.001 |
| Week 8 | st-PNS vs SON-PRF | −1.427 | 0.469 | −2.350 to −0.503 | 0.003 | 0.005 |
| Week 8 | st-PNS vs SON+SG-PRF | 0.614 | 0.477 | −0.326 to 1.554 | 0.199 | 0.199 |
| Week 8 | SON-PRF vs SON+SG-PRF | 2.041 | 0.462 | 1.132 to 2.950 | <0.001 | <0.001 |
| Week 12 | st-PNS vs SON-PRF | −1.355 | 0.555 | −2.447 to −0.263 | 0.015 | 0.030 |
| Week 12 | st-PNS vs SON+SG-PRF | 0.799 | 0.565 | −0.313 to 1.911 | 0.158 | 0.158 |
| Week 12 | SON-PRF vs SON+SG-PRF | 2.155 | 0.546 | 1.079 to 3.230 | <0.001 | <0.001 |
| Week 24 | st-PNS vs SON-PRF | −1.449 | 0.646 | −2.721 to −0.178 | 0.026 | 0.051 |
| Week 24 | st-PNS vs SON+SG-PRF | 1.063 | 0.658 | −0.232 to 2.358 | 0.107 | 0.107 |
| Week 24 | SON-PRF vs SON+SG-PRF | 2.512 | 0.636 | 1.260 to 3.765 | <0.001 | <0.001 |

Estimates were obtained from the baseline-adjusted linear mixed-effects model with participant-specific random intercepts and time random slopes. Holm adjustment was applied to the three pairwise comparisons separately at each follow-up visit. Time-specific comparisons were exploratory, and no additional adjustment was made across follow-up visits.

**Table S6. Overall model tests and pairwise comparisons of PHQ-9 trajectories using the linear mixed-effects model.**

| **Effect or comparison** | **χ²** | **df** | **P value** | **Adjusted P value** |
| --- | --- | --- | --- | --- |
| **Overall model tests** |  |  |  |  |
| Overall time-related effect | 52.071 | 15 | <0.001 | — |
| Group × time | 2.276 | 10 | 0.994 | — |
| Joint group effect | 5.931 | 12 | 0.920 | 0.920 |
| **Pairwise profile comparisons** |  |  |  |  |
| st-PNS vs SON-PRF | 1.577 | 6 | 0.954 | 1.000 |
| st-PNS vs SON+SG-PRF | 1.856 | 6 | 0.932 | 1.000 |
| SON-PRF vs SON+SG-PRF | 5.354 | 6 | 0.499 | 1.000 |

The model included participant-specific random intercepts and time random slopes and was adjusted for baseline PHQ-9. For the joint group effect, the adjusted P value was obtained by applying the Holm procedure across the three secondary outcomes (GAD-7, PHQ-9, and PSQI). For pairwise profile comparisons, Holm adjustment was applied to the three between-group comparisons within PHQ-9.

**Table S7. Time-specific pairwise comparisons of PHQ-9 scores from the linear mixed-effects model.**

| **Time** | **Comparison** | **Adjusted mean difference** | **SE** | **95% CI** | **Raw P** | **Holm-adjusted P** |
| --- | --- | --- | --- | --- | --- | --- |
| Week 1 | st-PNS vs SON-PRF | −0.282 | 0.384 | −1.038 to 0.473 | 0.462 | 0.816 |
| Week 1 | st-PNS vs SON+SG-PRF | 0.324 | 0.391 | −0.446 to 1.093 | 0.408 | 0.816 |
| Week 1 | SON-PRF vs SON+SG-PRF | 0.606 | 0.378 | −0.138 to 1.350 | 0.110 | 0.330 |
| Week 2 | st-PNS vs SON-PRF | −0.315 | 0.429 | −1.160 to 0.530 | 0.464 | 0.464 |
| Week 2 | st-PNS vs SON+SG-PRF | 0.529 | 0.437 | −0.332 to 1.390 | 0.227 | 0.455 |
| Week 2 | SON-PRF vs SON+SG-PRF | 0.844 | 0.423 | 0.012 to 1.677 | 0.047 | 0.141 |
| Week 4 | st-PNS vs SON-PRF | −0.534 | 0.499 | −1.516 to 0.448 | 0.285 | 0.571 |
| Week 4 | st-PNS vs SON+SG-PRF | 0.503 | 0.508 | −0.497 to 1.503 | 0.323 | 0.571 |
| Week 4 | SON-PRF vs SON+SG-PRF | 1.037 | 0.491 | 0.069 to 2.004 | 0.036 | 0.107 |
| Week 8 | st-PNS vs SON-PRF | −0.392 | 0.584 | −1.542 to 0.757 | 0.502 | 0.502 |
| Week 8 | st-PNS vs SON+SG-PRF | 0.714 | 0.594 | −0.456 to 1.885 | 0.230 | 0.461 |
| Week 8 | SON-PRF vs SON+SG-PRF | 1.107 | 0.575 | −0.025 to 2.239 | 0.055 | 0.166 |
| Week 12 | st-PNS vs SON-PRF | −0.434 | 0.678 | −1.769 to 0.901 | 0.523 | 0.523 |
| Week 12 | st-PNS vs SON+SG-PRF | 0.835 | 0.691 | −0.525 to 2.195 | 0.228 | 0.455 |
| Week 12 | SON-PRF vs SON+SG-PRF | 1.269 | 0.668 | −0.046 to 2.584 | 0.058 | 0.175 |
| Week 24 | st-PNS vs SON-PRF | −0.398 | 0.779 | −1.932 to 1.135 | 0.609 | 0.609 |
| Week 24 | st-PNS vs SON+SG-PRF | 0.841 | 0.793 | −0.721 to 2.402 | 0.290 | 0.580 |
| Week 24 | SON-PRF vs SON+SG-PRF | 1.239 | 0.767 | −0.271 to 2.750 | 0.107 | 0.322 |

Estimates were obtained from the baseline-adjusted linear mixed-effects model with participant-specific random intercepts and time random slopes. Holm adjustment was applied to the three pairwise comparisons separately at each follow-up visit. Time-specific comparisons were exploratory, and no additional adjustment was made across follow-up visits.

**Table S8. Multiplicity-adjusted analyses of the secondary outcomes**

**Panel A. Overall group-related effects across the three secondary outcomes**

| **Analysis** | **Outcome** | **Raw P** | **Holm-adjusted P across outcomes** |
| --- | --- | --- | --- |
| Unweighted LMM | GAD-7 | 0.003 | 0.006 |
| Unweighted LMM | PHQ-9 | 0.920 | 0.920 |
| Unweighted LMM | PSQI | <0.001 | <0.001 |
| Generalized overlap weighting | GAD-7 | 0.007 | 0.014 |
| Generalized overlap weighting | PHQ-9 | 0.890 | 0.890 |
| Generalized overlap weighting | PSQI | <0.001 | <0.001 |
| Stabilized IPTW | GAD-7 | 0.006 | 0.011 |
| Stabilized IPTW | PHQ-9 | 0.880 | 0.880 |
| Stabilized IPTW | PSQI | <0.001 | <0.001 |

Holm adjustment was applied to the three overall group-related P values for GAD-7.

**Panel B. Pairwise profile comparisons within each secondary outcome**

| **Analysis** | **Outcome** | **Comparison** | **Raw P** | **Holm-adjusted P within outcome** |
| --- | --- | --- | --- | --- |
| Unweighted LMM |  |  |  |  |
|  | GAD-7 | st-PNS vs SON-PRF | 0.025 | 0.049 |
|  | GAD-7 | st-PNS vs SON+SG-PRF | 0.774 | 0.774 |
|  | GAD-7 | SON-PRF vs SON+SG-PRF | <0.001 | <0.001 |
|  | PHQ-9 | st-PNS vs SON-PRF | 0.954 | 1.000 |
|  | PHQ-9 | st-PNS vs SON+SG-PRF | 0.932 | 1.000 |
|  | PHQ-9 | SON-PRF vs SON+SG-PRF | 0.499 | 1.000 |
|  | PSQI | st-PNS vs SON-PRF | <0.001 | <0.001 |
|  | PSQI | st-PNS vs SON+SG-PRF | 0.004 | 0.004 |
|  | PSQI | SON-PRF vs SON+SG-PRF | <0.001 | <0.001 |
| Generalized overlap weighting |  |  |  |  |
|  | GAD-7 | st-PNS vs SON-PRF | 0.107 | 0.214 |
|  | GAD-7 | st-PNS vs SON+SG-PRF | 0.714 | 0.714 |
|  | GAD-7 | SON-PRF vs SON+SG-PRF | <0.001 | 0.001 |
|  | PHQ-9 | st-PNS vs SON-PRF | 0.883 | 1.000 |
|  | PHQ-9 | st-PNS vs SON+SG-PRF | 0.936 | 1.000 |
|  | PHQ-9 | SON-PRF vs SON+SG-PRF | 0.543 | 1.000 |
|  | PSQI | st-PNS vs SON-PRF | <0.001 | <0.001 |
|  | PSQI | st-PNS vs SON+SG-PRF | 0.006 | 0.006 |
|  | PSQI | SON-PRF vs SON+SG-PRF | <0.001 | <0.001 |
| Stabilized IPTW |  |  |  |  |
|  | GAD-7 | st-PNS vs SON-PRF | 0.100 | 0.199 |
|  | GAD-7 | st-PNS vs SON+SG-PRF | 0.751 | 0.751 |
|  | GAD-7 | SON-PRF vs SON+SG-PRF | <0.001 | 0.001 |
|  | PHQ-9 | st-PNS vs SON-PRF | 0.885 | 1.000 |
|  | PHQ-9 | st-PNS vs SON+SG-PRF | 0.937 | 1.000 |
|  | PHQ-9 | SON-PRF vs SON+SG-PRF | 0.532 | 1.000 |
|  | PSQI | st-PNS vs SON-PRF | <0.001 | <0.001 |
|  | PSQI | st-PNS vs SON+SG-PRF | 0.011 | 0.011 |
|  | PSQI | SON-PRF vs SON+SG-PRF | <0.001 | <0.001 |

Note. For each analysis method, Holm adjustment was applied across the three overall group-related P values for GAD-7, PHQ-9, and PSQI.

**Table S9. Overall model tests and pairwise comparisons of PSQI trajectories using the linear mixed-effects model.**

| **Effect or comparison** | **χ²** | **df** | **P value** | **Adjusted P value** |
| --- | --- | --- | --- | --- |
| **Overall model tests** |  |  |  |  |
| Overall time-related effect | 318.701 | 15 | <0.001 | — |
| Group × time | 10.160 | 10 | 0.427 | — |
| Joint group effect | 99.742 | 12 | <0.001 | <0.001 |
| **Pairwise profile comparisons** |  |  |  |  |
| st-PNS vs SON-PRF | 31.148 | 6 | <0.001 | <0.001 |
| st-PNS vs SON+SG-PRF | 18.856 | 6 | 0.004 | 0.004 |
| SON-PRF vs SON+SG-PRF | 97.114 | 6 | <0.001 | <0.001 |

The model included participant-specific random intercepts and was adjusted for baseline PSQI. For the joint group effect, the adjusted P value was obtained by applying the Holm procedure across the three secondary outcomes (GAD-7, PHQ-9, and PSQI). For pairwise profile comparisons, Holm adjustment was applied to the three between-group comparisons within PSQI.

**Table S10. Time-specific pairwise comparisons of PSQI scores from the linear mixed-effects model.**

| **Time** | **Comparison** | **Adjusted mean difference** | **SE** | **95% CI** | **Raw P** | **Holm-adjusted P** |
| --- | --- | --- | --- | --- | --- | --- |
| Week 1 | st-PNS vs SON-PRF | −0.165 | 0.558 | −1.263 to 0.934 | 0.768 | 0.768 |
| Week 1 | st-PNS vs SON+SG-PRF | 0.851 | 0.568 | −0.266 to 1.969 | 0.135 | 0.270 |
| Week 1 | SON-PRF vs SON+SG-PRF | 1.016 | 0.549 | −0.065 to 2.098 | 0.065 | 0.196 |
| Week 2 | st-PNS vs SON-PRF | −0.757 | 0.558 | −1.855 to 0.341 | 0.176 | 0.176 |
| Week 2 | st-PNS vs SON+SG-PRF | 1.047 | 0.568 | −0.071 to 2.165 | 0.066 | 0.133 |
| Week 2 | SON-PRF vs SON+SG-PRF | 1.804 | 0.549 | 0.722 to 2.885 | 0.001 | 0.003 |
| Week 4 | st-PNS vs SON-PRF | −1.242 | 0.558 | −2.341 to −0.144 | 0.027 | 0.053 |
| Week 4 | st-PNS vs SON+SG-PRF | 1.266 | 0.568 | 0.148 to 2.384 | 0.027 | 0.053 |
| Week 4 | SON-PRF vs SON+SG-PRF | 2.508 | 0.549 | 1.427 to 3.590 | <0.001 | <0.001 |
| Week 8 | st-PNS vs SON-PRF | −1.473 | 0.558 | −2.571 to −0.374 | 0.009 | 0.018 |
| Week 8 | st-PNS vs SON+SG-PRF | 1.081 | 0.568 | −0.037 to 2.199 | 0.058 | 0.058 |
| Week 8 | SON-PRF vs SON+SG-PRF | 2.554 | 0.549 | 1.472 to 3.635 | <0.001 | <0.001 |
| Week 12 | st-PNS vs SON-PRF | −1.650 | 0.558 | −2.748 to −0.552 | 0.003 | 0.007 |
| Week 12 | st-PNS vs SON+SG-PRF | 1.223 | 0.568 | 0.105 to 2.341 | 0.032 | 0.032 |
| Week 12 | SON-PRF vs SON+SG-PRF | 2.873 | 0.549 | 1.792 to 3.955 | <0.001 | <0.001 |
| Week 24 | st-PNS vs SON-PRF | −1.797 | 0.558 | −2.896 to −0.699 | 0.001 | 0.003 |
| Week 24 | st-PNS vs SON+SG-PRF | 0.654 | 0.568 | −0.464 to 1.772 | 0.250 | 0.250 |
| Week 24 | SON-PRF vs SON+SG-PRF | 2.451 | 0.549 | 1.370 to 3.533 | <0.001 | <0.001 |

Estimates were obtained from the baseline-adjusted linear mixed-effects model with participant-specific random intercepts. Holm adjustment was applied to the three pairwise comparisons separately at each follow-up visit. Time-specific comparisons were exploratory, and no additional adjustment was made across follow-up visits.

**Table S11. Covariate balance before and after propensity score weighting**

| **Covariate** | **Unweighted maximum \|SMD\|** | **Overlap-weighted maximum \|SMD\|** | **Stabilized IPTW maximum \|SMD\|** |
| --- | --- | --- | --- |
| Age | 0.134 | 0.009 | 0.017 |
| Sex | 0.311 | 0.049 | 0.036 |
| Affected side | 0.303 | 0.009 | 0.009 |
| Disease duration | 0.162 | 0.062 | 0.073 |
| History of antiviral therapy | 0.240 | 0.023 | 0.014 |
| Baseline NRS | 0.206 | 0.047 | 0.033 |
| Baseline GAD-7 | 0.088 | 0.021 | 0.024 |
| Baseline PHQ-9 | 0.091 | 0.074 | 0.086 |
| Baseline PSQI | 0.183 | 0.003 | 0.009 |

Note. Values are the maximum absolute standardized mean difference (SMD) across the three pairwise group comparisons. The propensity score model included the prespecified pretreatment demographic and clinical variables and the baseline value of each outcome. An absolute SMD <0.10 was considered acceptable balance.

**Table S12. Longitudinal outcome analyses after generalized overlap weighting**

| **Outcome** | **Test family** | **Effect or comparison** | **χ²** | **df** | **Raw P** | **Holm-adjusted P** |
| --- | --- | --- | --- | --- | --- | --- |
| NRS |  |  |  |  |  |  |
|  | Overall | Overall time-related effect‡ | 124.931 | 15 | <0.001 | — |
|  | Overall | Group × time | 17.562 | 10 | 0.063 | — |
|  | Overall | Overall group-related effect† | 26.143 | 12 | 0.010 | — |
|  | Pairwise profile | st-PNS vs SON-PRF | 16.220 | 6 | 0.013 | 0.038 |
|  | Pairwise profile | st-PNS vs SON+SG-PRF | 7.084 | 6 | 0.313 | 0.313 |
|  | Pairwise profile | SON-PRF vs SON+SG-PRF | 13.720 | 6 | 0.033 | 0.066 |
| GAD-7 |  |  |  |  |  |  |
|  | Overall | Overall time-related effect‡ | 225.106 | 15 | <0.001 | — |
|  | Overall | Group × time | 22.525 | 10 | 0.013 | — |
|  | Overall | Overall group-related effect† | 27.266 | 12 | 0.007 | — |
|  | Pairwise profile | st-PNS vs SON-PRF | 10.455 | 6 | 0.107 | 0.214 |
|  | Pairwise profile | st-PNS vs SON+SG-PRF | 3.724 | 6 | 0.714 | 0.714 |
|  | Pairwise profile | SON-PRF vs SON+SG-PRF | 24.272 | 6 | <0.001 | 0.001 |
| PHQ-9 |  |  |  |  |  |  |
|  | Overall | Overall time-related effect‡ | 58.424 | 15 | <0.001 | — |
|  | Overall | Group × time | 3.127 | 10 | 0.978 | — |
|  | Overall | Overall group-related effect† | 6.487 | 12 | 0.890 | — |
|  | Pairwise profile | st-PNS vs SON-PRF | 2.363 | 6 | 0.883 | 1.000 |
|  | Pairwise profile | st-PNS vs SON+SG-PRF | 1.810 | 6 | 0.936 | 1.000 |
|  | Pairwise profile | SON-PRF vs SON+SG-PRF | 5.004 | 6 | 0.543 | 1.000 |
| PSQI |  |  |  |  |  |  |
|  | Overall | Overall time-related effect‡ | 314.589 | 15 | <0.001 | — |
|  | Overall | Group × time | 9.873 | 10 | 0.452 | — |
|  | Overall | Overall group-related effect† | 93.954 | 12 | <0.001 | — |
|  | Pairwise profile | st-PNS vs SON-PRF | 27.510 | 6 | <0.001 | <0.001 |
|  | Pairwise profile | st-PNS vs SON+SG-PRF | 18.027 | 6 | 0.006 | 0.006 |
|  | Pairwise profile | SON-PRF vs SON+SG-PRF | 91.032 | 6 | <0.001 | <0.001 |

Note. Generalized overlap weights were used; all 269 participants were retained. Weighted generalized estimating equations with an exchangeable working correlation structure and robust sandwich standard errors were fitted to the six follow-up measurements. Models included treatment group, categorical follow-up time, the group-by-time interaction, and the corresponding baseline score. Pairwise profile P values were Holm-adjusted across the three comparisons within each outcome. Cross-outcome multiplicity adjustment for GAD-7, PHQ-9, and PSQI is reported in Table S8. †Joint test of all group and group-by-time terms. ‡Joint test of all time and group-by-time terms.

**Table S13. Longitudinal outcome analyses using stabilized inverse probability of treatment weighting**

| **Outcome** | **Test family** | **Effect or comparison** | **χ²** | **df** | **Raw P** | **Holm-adjusted P** |
| --- | --- | --- | --- | --- | --- | --- |
| NRS |  |  |  |  |  |  |
|  | Overall | Overall time-related effect‡ | 121.621 | 15 | <0.001 | — |
|  | Overall | Group × time | 18.375 | 10 | 0.049 | — |
|  | Overall | Overall group-related effect† | 26.694 | 12 | 0.009 | — |
|  | Pairwise profile | st-PNS vs SON-PRF | 16.414 | 6 | 0.012 | 0.035 |
|  | Pairwise profile | st-PNS vs SON+SG-PRF | 7.432 | 6 | 0.283 | 0.283 |
|  | Pairwise profile | SON-PRF vs SON+SG-PRF | 13.943 | 6 | 0.030 | 0.061 |
| GAD-7 |  |  |  |  |  |  |
|  | Overall | Overall time-related effect‡ | 230.188 | 15 | <0.001 | — |
|  | Overall | Group × time | 23.039 | 10 | 0.011 | — |
|  | Overall | Overall group-related effect† | 27.929 | 12 | 0.006 | — |
|  | Pairwise profile | st-PNS vs SON-PRF | 10.656 | 6 | 0.100 | 0.199 |
|  | Pairwise profile | st-PNS vs SON+SG-PRF | 3.446 | 6 | 0.751 | 0.751 |
|  | Pairwise profile | SON-PRF vs SON+SG-PRF | 24.897 | 6 | <0.001 | 0.001 |
| PHQ-9 |  |  |  |  |  |  |
|  | Overall | Overall time-related effect‡ | 58.225 | 15 | <0.001 | — |
|  | Overall | Group × time | 2.807 | 10 | 0.986 | — |
|  | Overall | Overall group-related effect† | 6.645 | 12 | 0.880 | — |
|  | Pairwise profile | st-PNS vs SON-PRF | 2.348 | 6 | 0.885 | 1.000 |
|  | Pairwise profile | st-PNS vs SON+SG-PRF | 1.803 | 6 | 0.937 | 1.000 |
|  | Pairwise profile | SON-PRF vs SON+SG-PRF | 5.095 | 6 | 0.532 | 1.000 |
| PSQI |  |  |  |  |  |  |
|  | Overall | Overall time-related effect‡ | 308.677 | 15 | <0.001 | — |
|  | Overall | Group × time | 9.832 | 10 | 0.455 | — |
|  | Overall | Overall group-related effect† | 92.612 | 12 | <0.001 | — |
|  | Pairwise profile | st-PNS vs SON-PRF | 27.020 | 6 | <0.001 | <0.001 |
|  | Pairwise profile | st-PNS vs SON+SG-PRF | 16.536 | 6 | 0.011 | 0.011 |
|  | Pairwise profile | SON-PRF vs SON+SG-PRF | 89.537 | 6 | <0.001 | <0.001 |

Note. Stabilized inverse probability of treatment weights were used; all 269 participants were retained. Weighted generalized estimating equations with an exchangeable working correlation structure and robust sandwich standard errors were fitted to the six follow-up measurements. Models included treatment group, categorical follow-up time, the group-by-time interaction, and the corresponding baseline score. Pairwise profile P values were Holm-adjusted across the three comparisons within each outcome. Cross-outcome multiplicity adjustment for GAD-7, PHQ-9, and PSQI is reported in Table S8. †Joint test of all group and group-by-time terms. ‡Joint test of all time and group-by-time terms.

**Table S14. Weighted clinical response rates and between-group comparisons**

| **Method** | **Time** | **st-PNS** | **SON-PRF** | **SON+SG-PRF** | **Overall P** | **st-PNS vs SON-PRF** | **st-PNS vs SON+SG-PRF** | **SON-PRF vs SON+SG-PRF** |
| --- | --- | --- | --- | --- | --- | --- | --- | --- |
| Overlap weighting |  |  |  |  |  |  |  |  |
|  | Week 1 | 90.3% | 87.7% | 90.8% | 0.755 | 1.000 | 1.000 | 1.000 |
|  | Week 2 | 96.9% | 83.4% | 94.8% | 0.005 | 0.016 | 0.467 | 0.039 |
|  | Week 4 | 94.6% | 77.6% | 91.2% | 0.002 | 0.007 | 0.378 | 0.028 |
|  | Week 8 | 91.5% | 70.5% | 86.4% | 0.001 | 0.004 | 0.294 | 0.021 |
|  | Week 12 | 88.6% | 70.1% | 81.9% | 0.013 | 0.017 | 0.236 | 0.128 |
|  | Week 24 | 85.2% | 67.6% | 77.4% | 0.036 | 0.035 | 0.283 | 0.283 |
| Stabilized IPTW |  |  |  |  |  |  |  |  |
|  | Week 1 | 90.2% | 87.8% | 90.8% | 0.782 | 1.000 | 1.000 | 1.000 |
|  | Week 2 | 96.8% | 83.5% | 94.9% | 0.005 | 0.021 | 0.507 | 0.036 |
|  | Week 4 | 94.6% | 77.7% | 90.9% | 0.003 | 0.007 | 0.333 | 0.034 |
|  | Week 8 | 91.5% | 70.4% | 86.0% | 0.002 | 0.004 | 0.263 | 0.025 |
|  | Week 12 | 88.0% | 69.9% | 81.5% | 0.021 | 0.028 | 0.273 | 0.144 |
|  | Week 24 | 84.9% | 67.3% | 76.8% | 0.043 | 0.043 | 0.312 | 0.312 |

Note. Clinical response was defined as a reduction in NRS score of at least 50% from baseline. Values are weighted percentages. Overall and pairwise comparisons were obtained from weighted binomial generalized estimating equations with robust sandwich standard errors. Pairwise P values were Holm-adjusted across the three comparisons within each follow-up visit and were not additionally adjusted across visits. IPTW, inverse probability of treatment weighting; NRS, Numerical Rating Scale.

**Figure S1. Covariate balance before and after propensity score weighting**
